# Combined Effects of Allelopathic Polyphenols on *Microcystis aeruginosa* and Response of Different Chlorophyll Fluorescence Parameters

**DOI:** 10.3389/fmicb.2020.614570

**Published:** 2020-12-01

**Authors:** Suzhen Huang, Junying Zhu, Lu Zhang, Xue Peng, Xinyi Zhang, Fangjie Ge, Biyun Liu, Zhenbin Wu

**Affiliations:** ^1^State Key Laboratory of Freshwater Ecology and Biotechnology, Institute of Hydrobiology, Chinese Academy of Sciences, Wuhan, China; ^2^University of Chinese Academy of Sciences, Beijing, China; ^3^Sinopec Research Institute of Petroleum Processing, Beijing, China

**Keywords:** allelopathy, polyphenols, combined effect, cyanobacteria, cell densities, chlorophyll fluorescence parameters

## Abstract

Polyphenols are allelochemicals secreted by aquatic plants that effectively control cyanobacteria blooms. In this study, sensitive response parameters (including CFPs) of *Microcystis aeruginosa* were explored under the stress of different polyphenols individually and their combination. The combined effects on *M. aeruginosa* were investigated based on the most sensitive parameter and cell densities. For pyrogallic acid (PA) and gallic acid (GA), the sensitivity order of parameters based on the EC_50_ values (from 0.73 to 3.40 mg L^–1^ for PA and from 1.05 to 2.68 mg L^–1^ for GA) and the results of the hierarchical cluster analysis showed that non-photochemical quenching parameters [NPQ, *q*_N_, *q*_N(rel)_ and *q*_CN_] > photochemical quenching parameters [YII, *q*_P_, *q*_P(rel)_ and *q*_L_] or others [*F*_v_/*F*_m_, *F’*_v_*/F’*_m_, *q*_TQ_ and UQF_(rel)_] > cell densities. CFPs were not sensitive to ellagic acid (EA) and (+)-catechin (CA). The sensitivity order of parameters for *M. aeruginosa* with PA-GA mixture was similar to that under PA and GA stress. The quantitative (Toxicity Index, TI) and qualitative (Isobologram representation) methods were employed to evaluate the combined effects of PA, GA, and CA on *M. aeruginosa* based on cell densities and NPQ. TI values based on the EC_50 cells_ suggested the additive effects of binary and multiple polyphenols, but synergistic and additive effects according to the EC_50 NPQ_ (varied from 0.16 to 1.94). In terms of NPQ of *M. aeruginosa*, the binary polyphenols exhibited synergistic effects when the proportion of high toxic polyphenols PA or GA was lower than 40%, and the three polyphenols showed a synergistic effect only at the ratio of 1:1:1. Similar results were also found by isobologram representation. The results showed that increasing the ratio of high toxic polyphenols would not enhance the allelopathic effects, and the property, proportion and concentrations of polyphenols played an important role in the combined effects. Compared with cell densities, NPQ was a more suitable parameter as evaluating indicators in the combined effects of polyphenols on *M. aeruginosa*. These results could provide a method to screen the allelochemicals of polyphenols inhibiting cyanobacteria and improve the inhibitory effects by different polyphenols combined modes.

## Introduction

Polyphenols are well known secondary metabolites in the plant kingdom not only because of their possible health benefits arising from such as their antioxidant activity, antimicrobial capacity and anticancer property (Belščak-Cvitanović et al., 2018; Bouarab-Chibane et al., 2019; Álvarez-Martínez et al., 2020), but also because of their allelopathic inhibition on nuisance phytoplankton in the aquatic ecosystem (Gao et al., 2017; Tan et al., 2019). Many polyphenols, such as pyrogallic acid (PA), gallic acid (GA), ellagic acid (EA), and (+)-catechin (CA), have been identified in aquatic plants (Nakai et al., 2000; Olate-Gallegos et al., 2019; Zolotareva et al., 2019), and confirmed evidently to have allelopathic inhibition against various cyanobacteria (Huang et al., 2016; Techer et al., 2016; Yu et al., 2019). Polyphenols from natural biomaterials have received more attention as alternatives for emergency control of cyanobacteria bloom because of their algae-inhibiting effects, which is specific to cyanobacteria, biodegradable and less toxic effect to other aquatic organisms (Mohamed, 2017; Tan et al., 2019).

Among polyphenols, such as pyrogallic acid (PA), performs a strong inhibitory activity against *M. aeruginosa*, which is one of the common dominant species of cyanobacteria blooms and the most serious all over the world (Qin et al., 2018; Benayache et al., 2019). Polyphenols exert inhibitory effects on cyanobacteria by direct oxidative damage for causing overproduction of free radicals, or leading to the programmed cell death for inducing signal molecules changes, such as the nitric oxide and hydrogen peroxide (Wang et al., 2013; Lu et al., 2017). Although polyphenols have obvious algae-inhibiting activity, how to explain individual allelochemical with the higher 50% effective concentrations (EC_50_ values) and much lower concentration released by aquatic plants is still a gap. Nakai et al. (2000) reported that the 72 h EC_50_ values of PA, GA, EA and CA were 0.65, 1.0, 5.1, and 5.5 mg L^–1^ for *M. aeruginosa* (NIES-87), respectively. However, the concentrations of polyphenols released to the culture water were μg L^–1^ levels (Hilt et al., 2006). It is all known that aquatic plants can release different types of allelochemical into the environment, and their contents and compositions are different for distinct aquatic plants (Tan et al., 2019). There is growing evidence that the combined effects of allelochemicals play an important role in the allelopathy of aquatic plants, and may help to reveal the mechanism of allelopathy in natural aquatic ecosystems (Gao et al., 2015, 2017). A variety of allelochemicals, which coexist in the same environment, show the combined effects, such as additive or synergistic effects. Previous research indicated that the combined effects of allelochemicals were related to their property, proportion and concentrations (Gao et al., 2015; Zuo et al., 2016b). The polyphenols with the proportion identified in the *Myriophyllum spicatum-*cultured solution presented a synergistic effect to inhibit *M. aeruginosa* growth (Zhu et al., 2010). Synergistic effects significantly reduced the concentration contribution of a single allelochemical to the EC_50_ value. Further study found that the effects of individual polyphenols such as PA and GA on the 72 h EC_50_ values of *M. aeruginosa* were different according to the cell densities, NPQ and YII parameters (Zhu et al., 2010). The value of EC_50 cells_ was far higher than that of EC_50 NPQ_ and EC_50 YII_. There were also differences between EC_50 NPQ_ and EC_50 YII_.

Cyanobacteria photosynthetic systems are the main target sites of polyphenols, which have been used to indicate the response of photosynthesis to environmental stresses (Bussotti et al., 2020; Zhu et al., 2021). PA and GA can inhibit the photosystem II (PSII) activity of cyanobacteria, eventually lead to the suppression of the photosynthetic system (Zhu et al., 2010). Chlorophyll is one of the main photosynthetic pigments and the foundation of algae photosynthesis (Zhu et al., 2021). Most of the light energy absorbed by chlorophyll is used for photosynthesis, heat (non-photochemical quenching-NPQ) and chlorophyll fluorescence (Hanelt, 2018; Zulfugarov et al., 2018). The change of photosynthesis can cause the corresponding change of fluorescence emission because of the mutual competition for energy among these three ways (Zulfugarov et al., 2018). The fluorescence mainly reflects the efficiency of the photochemical energy conversion of PSII reaction centers (Murchie and Lawson, 2013). Different chlorophyll fluorescence parameters (CFPs) suggest different changes of PSII, which contains photochemical quenching parameters [e.g., YII, *q*_P_, *q*_P(rel)_ and *q*_L_]; non-photochemical quenching parameters (e.g., NPQ, *q*_N_, *q*_N(rel)_ and *q*_CN_); others [e.g. *F*_v_/*F*_m_, *F’*_v_*/F’*_m_, *q*_TQ_ and UQF_(rel)_]. Different CFPs may present different sensitivities under the same stress (Wang et al., 2016). When *Scenedesmus obliquus* was exposed to isoproturon [3-(4-isopropylphenyl)-1,1-dimethylurea], the order of parameters sensitivity was UQF_(rel)_ > YII > *q*_P_ > cell densities > *q*_P(rel)_ (Dewez et al., 2008). *F*_v_/*F*_m_ and *F’*_v_*/F’*_m_ were more sensitive parameters than the cell densities in the toxic test of allelochemical ferulic acid and NH_4+_ toxicity (Wang et al., 2016; Berg et al., 2017). Gan et al. (2019) studied the effects of lead concentrations on the five CFPs of marine microalgae *Nitzschia closterium*, and found the PSII electron flux per unit volume and effective quantum yield of PSII photochemical energy conversion were the most sensitive CFPs. The varied sensitivities of different CFPs may relate to the different toxicity mechanisms of tested compounds. Whether there is a more sensitive CFPs parameters to characterize the inhibitory effect of different polyphenols (independently or jointly) on cyanobacteria?

In this research, the sensitivities of different CFPs were studied based on the 72 h EC_50_ values of PA, GA, EA, and CA on *M. aeruginosa* including 12 kinds of CFPs and cell densities. Besides, the combined effects of three polyphenols PA, GA, and CA were studied with a series of different proportions. The 72 EC_50_ values of them were calculated based on cell densities and NPQ. The combined effects were analyzed according to quantitative and qualitative methods. Our aims are to (1) screen sensitive response parameter to individual polyphenols stress, (2) determine sensitive response parameters to combined polyphenols, (3) and find the combined effects of polyphenols with different properties, proportion based on cell densities and the most sensitive parameter. These results could provide a method to screen the allelochemicals of polyphenols inhibiting cyanobacteria and improve the inhibitory effects by different polyphenols combined modes.

## Materials and Methods

### Culture Conditions

*M. aeruginosa* (toxic FACHB 905) was provided by the Freshwater Algae Culture Collection of the Institute of Hydrobiology, the Chinese Academy of Sciences. It was axenically cultivated in MIII medium at 25 ± 2°C in an incubator with a light/dark cycle of 12:12 h and an irradiance of 57 μmol photons m^–2^s^–1^, and manually shaken three times every day. In this study, *M. aeruginosa* in the exponential growth phase was used.

### Polyphenols Chemicals

The polyphenols PA (99.5%, Chemservice), GA (>96.0%, ACROS), EA (≥96.0%, Fluka) and CA (98%, TCI) were commercially obtained. Dimethyl sulfoxide (DMSO, > 98%) was of analytical grade.

### Bioassays of Independent and Mixed Polyphenols

The dose-response relationships between polyphenols and *M. aeruginosa* were performed. The initial algae density was 1 × 10^6^ cells mL^–1^. The final concentrations of independent polyphenols in the test solution were, respectively, 0.5, 1, 2.5, 5, 7.5 and 10 mg L^–1^ for PA and GA, and 2.5, 5, 7.5, 10, 20, and 50 mg L^–1^ for EA and CA. PA, GA, and CA were selected to study the combined effects of polyphenols. The component ratios were 1:4, 2:3, 1:1, 3:2, and 4:1 in binary polyphenols, and 1:1:3, 1:2:2, 1:3:1, 1:1:1, 2:1:2, 2:2: 1, 3:1:1 in three polyphenols. The final concentrations of polyphenols in the test solution were 0.5, 1, 2.5, 5, 7.5, 10, and 15 mg L^–1^. The 50% inhibition concentrations of every polyphenol based on the CFPs and cell densities of *M. aeruginosa* were determined after exposure for 72 h. The stock solutions of polyphenols were prepared with DMSO, which in the test solution was lower than 0.3% (v/v). And the test results indicated that adding DMSO did not affect the photosynthetic activity and cells.

The inhibition percentage of PA and GA mixture on *M. aeruginosa* were determined according to CFPs and cell densities after 24 h. The proportion of mixture was: PA: GA/1:1. The final concentration of PA-GA in the test solution was 2.5 mg L^–1^. And the inhibition percentage was calculated by the following equation:

Inhibition percentage = 100 (X_control_ – X_treatment_)/X_control_

where X_control_ represented the different parameters of *M. aeruginosa* in control, and X_treatment_ was the corresponding parameters of *M. aeruginosa* in treatment.

### Determination of Cell Densities and Chlorophyll Fluorescence Parameters

The cell densities of *M. aeruginosa* were examined with a light microscope and hemocytometer (Huang et al., 2016). The CFPs were determined by pulse amplitude modulated fluorometer (Phyto-PAM Walz, Effeltrich, Germany). The details could be obtained in Campbell et al. (1998) and Mallick and Mohn (2003). *F*_o_ (minimum fluorescence), *F’*_o_ (minimum fluorescence in the light-adapted state), *F*_s_ (steady-state fluorescence), *F’*_m_ (maximal fluorescence in the light-adapted state) and *F*_m_ (maximal fluorescence) were determined. *F*_o_ was determined after a dark-adapted for 5 min. *F’*_o_ was calculated according to the formula in Oxborough and Baker (1997). *F*_s_ was achieved by illuminating for 3 min with actinic light at 64 μmol photons m^–2^s^–1^, a similar light intensity to the cultivation during the experiment. The time of actinic light exposure was optimized before starting. Then a saturating light pulse was given to get *F’*_m_. *F*_m_ was determined in the presence of 10 mM 3-(3,4-dichlorophenyl)-1,1-dimethylurea (DCMU) due to state transition (Campbell et al., 1998). The 12 kinds of CFPs calculated from slow chlorophyll fluorescence induction kinetics were listed in Table 1 (Roháček and Barták, 1999; Kramer et al., 2004; Juneau et al., 2005).

**TABLE 1:** The equations of chlorophyll fluorescence parameters used in this study.

	Parameters	Equations
Photochemical quenching	YII	(*F’*_m_ – *F*_s_)/*F’*_m_
parameters	*q*_P_	(*F’*_m_ – *F*_s_)/(*F’*_m_ – *F’*_0_)
	*q*_P(rel)_	(*F’*_m_ – *F*_s_)/(*F*_m_ – *F’*_0_)
	*q*_L_	(*F’*_m_ – *F*_s_)/(*F’*_m_ – *F’*_0_)(*F’*_0_/*F*_s_)
Non-photochemical quenching	NPQ	(*F*_m_ – *F’*_m_)/*F’*_m_
parameters	*q*_N_	1 – (*F’*_m_ – *F’*_0_)/(*F*_m_ – *F*_0_)
	*q*_N(rel)_	(*F*_m_ – *F’*_m_)/(*F*_m_ – *F’*_0_)
	*q*_CN_	(*F*_m_ – *F’*_m_)/*F*_m_
Others	*q*_TQ_	(*F*_m_ – *F*_s_)/*F*_m_
	*F*_v_/*F*_m_	(*F*_m_ – *F*_0_)/*F*_m_
	*F’*_v_*/F’*_m_	(*F’*_m_ – *F’*_0_)/*F’*_m_
	UQF_(rel)_	(*F*_s_ – *F’*_0_)/(*F*_m_ – *F’*_0_)

### Estimation of Combined Effects

Toxicity index (TI) and isobologram representation were employed to evaluate the combined effects of polyphenols on target organisms in this study.

#### TI Model

TI was calculated by the following equation (Gao et al., 2015):

TI = Σ(*C*mix_A_/EC_50A_).

Where *C*mix_A_ was the concentration of component A at the EC_50_ value of the mixture (mg L^–1^), and EC_50A_ was the EC_50_ value of component A measured individually (mg L^–1^). The additional effect was characterized by 0.5 < TI < 2. The value of TI > 2 represented antagonism and TI < 0.5 indicated synergism.

#### Toxic Unit and Isobolograms

The theory of toxic unit (TU) was introduced by Brown (1968) and Sprague (1970) and calculated by the equation:

TU_A_ = *C*mix_A_/EC_50A_.

EC_50A_ was the effective concentration required to gain 50% inhibition of component A individually, and *C*mix_A_ corresponded to EC_50_ of A in the mixture. Then TU values of the binary mixtures were plotted on an isobologram, which characterizes the combined effects of binary mixtures on target organisms. The additivity line (linear equation: X + Y = 1.0) was a straight line joining TU of every single component, which represented the additive effect of the mixtures (Kortenkamp and Altenburger, 1998). In this research, Deneer’s method was employed to explain the endpoint of combined effects (Deneer, 2000). The additivity effects were the region between the up additivity line (linear equation: X + Y = 2.0) and low additivity line (linear equation: X + Y = 0.5). When an isobole was below the low additivity line, the components presented synergistic effects, whereas, it is depicted as an antagonistic effect above the up additivity line (Calamari and Alabaster, 1980).

### Statistical Analysis

The 50% inhibition concentrations based on CFPs and cell densities of *M. aeruginosa* were calculated from probit regression generated by SPSS 13.0 for Windows. Tukey’s honestly significant difference test and *T*-test were used to test the statistical significance of the differences between controls and treatments. The dendrograms of hierarchical cluster analysis for CFPs and cell densities were done by SPSS 13.0 software. Ward’s method was applied and Squared Euclidean Distance was chosen as a measurement for hierarchical cluster analysis.

## Results

### Parameters of EC_50_ and Its Order of Individual Polyphenol on *M. aeruginosa*

The 72 h EC_50_ values of PA based on CFPs and cell densities of *M. aeruginosa* varied from 0.73 to 3.40 mg L^–1^ for PA, 1.05 to 2.68 mg L^–1^ for GA, 17.95 to 52.38 mg L^–1^ for EA, and 17.95 to 52.38 mg L^–1^ for CA (Table 2). As displayed in Table 2, the order of parameters according to the EC_50_ values was: *q*_CN_ < NPQ < *q*_N_ < *q*_N(rel)_ < UQF_(rel)_ < YII < *q*_TQ_ < *F*_v_/*F*_m_ < *F’*_v_*/F’*_m_ < *q*_P(rel)_ < cell densities < *q*_P_ < *q*_L_ for PA, NPQ < *q*_N_ < *q*_CN_ < *q*_N(rel)_ < UQF_(rel)_ < YII < *q*_P(rel)_ < *q*_L_ < *q*_TQ_ < *q*_P_ < *F*_v_/*F*_m_ < *F’*_v_*/F’*_m_ < cell densities for GA, *q*_CN_ < NPQ < UQF_(rel)_ < *q*_TQ_ < YII < *q*_P(rel)_ < *q*_L_ < cell densities < *q*_P_ < *F*_v_/*F*_m_ < *q*_N_ < *F’*_v_*/F’*_m_ < *q*_N(rel)_ for EA, and cell densities < NPQ < *q*_TQ_ < YII < *F*_v_/*F*_m_ < *F’*_v_*/F’*_m_ < UQF_(rel)_ < *q*_CN_ < *q*_N_ < *q*_P(rel)_ < *q*_L_ < *q*_N(rel)_ < *q*_P_ for CA. CFPs were more sensitive than cell densities in PA and GA, but which was not found in EA and CA. Besides, the parameters sensitivity order of PA was consistent with that of GA, and those of EA were close to CA. Therefore, the four polyphenols were divided into two subsets for further hierarchical cluster analysis.

**TABLE 2:** The 72 h EC_50_ values of individual allelopathic polyphenol based on chlorophyll fluorescence parameters and cell densities of *M. aeruginosa*.

Unit: mg/L								

	PA	95% CI	GA	95% CI	EA	95% CI	CA	95% CI
Cell densities	3.38^a^	3.01–3.75	2.68^a^	2.48–2.89	38.12^a^	33.19–43.04	10.05^a^	9.12–10.98
YII	1.97^be^	1.57–2.38	1.66^bd^	1.35–1.97	34.50^ae^	29.94–39.05	15.12^ab^	13.03–17.20
*q*_P_	3.40^ad^	2.76–4.03	2.02^bf^	1.68–2.35	39.58^af^	34.02–45.15	27.03^c^	24.18–29.87
*q*_P(rel)_	2.47^df^	2.17–2.76	1.88^bef^	1.55–2.22	37.55^a^	34.40–40.70	24.15^cf^	20.69–27.61
*q*_L_	3.40^ad^	2.98–3.81	1.90^bef^	1.79–2.00	37.57^a^	34.24–40.90	24.20^c^	21.48–26.91
NPQ	0.87^c^	0.57–1.18	1.05^c^	0.88–1.21	18.73^b^	16.98–20.48	11.67^aeg^	9.33–14.00
*q*_N_	1.03^ce^	0.73–1.32	1.30^cd^	1.11–1.49	42.28^ac^	37.48–47.08	22.75^cdf^	18.92–26.57
*q*_N(rel)_	1.06^c^	0.92–1.21	1.37^cde^	1.11–1.63	52.38^d^	45.47–59.28	26.26^c^	21.42–31.11
*q*_CN_	0.73^c^	0.50–0.97	1.34^cd^	1.14–1.54	17.95^b^	15.95–19.94	18.83^bdf^	15.88–21.77
*q*_TQ_	2.18^bf^	1.84–2.52	1.92^b^	1.61–2.23	26.45^be^	22.96–29.93	15.11^ab^	13.13–17.10
*F*_v_/*F*_m_	2.28^bf^	1.87–2.68	2.14^ab^	1.79–2.49	41.03^ac^	31.65–50.41	15.30^ab^	13.47–17.27
*F’*_v_*/F’*_m_	2.32^bf^	1.96–2.67	2.19^ab^	1.77–2.62	48.24^cdf^	43.71–52.77	15.41^be^	13.13–17.69
UQF_(rel)_	1.88^b^	1.71–2.06	1.51^cdf^	1.28–1.74	21.74^b^	18.22–25.26	16.87^bg^	14.35–19.38

*Values were means and 95% confidence interval (95% CI) (n = 6). The significant differences were signed with different superscript letters (a–g) in the same column (P < 0.05).*

**FIGURE 1:**
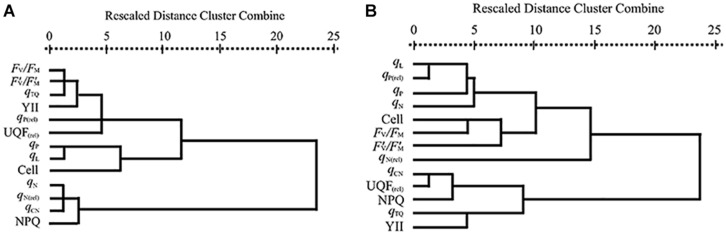
Dendrogram of hierarchical cluster analysis according to 72 h EC_50_ values of **(A)** PA and GA and **(B)** EA and CA to *M. aeruginosa* based on CFPs and cell densities. Cell means cell densities.

The results of hierarchical cluster analysis were presented in Figure 1. As shown in Figure 1A, the parameters of PA and GA were divided into three main clusters: (1) *q*_P_, *q*_L_ and cell densities; (2) *F*_v_/*F*_m_, *F’*_v_*/F’*_m_, *q*_TQ_, YII, *q*_P(rel)_ and UQF_(rel)_; (3) *q*_N_, *q*_N(rel)_, *q*_CN_ and NPQ. Figure 1B displayed that three main clusters were gained from the parameters of EA and CA: (1) *q*_CN_, UQF_(rel)_, NPQ, *q*_TQ_ and YII; (2) cell densities, *F*_v_/*F*_m_, *F’*_v_*/F’*_m_ and *q*_N(rel)_; (3) *q*_L_, *q*_P(rel)_, *q*_P_ and *q*_N_. The clusters based on hierarchical cluster analysis agreed well with the results obtained from the above EC_50_ values.

### The Response Parameters and Its Order of PA-GA Mixture on *M. aeruginosa*

**FIGURE 2:**
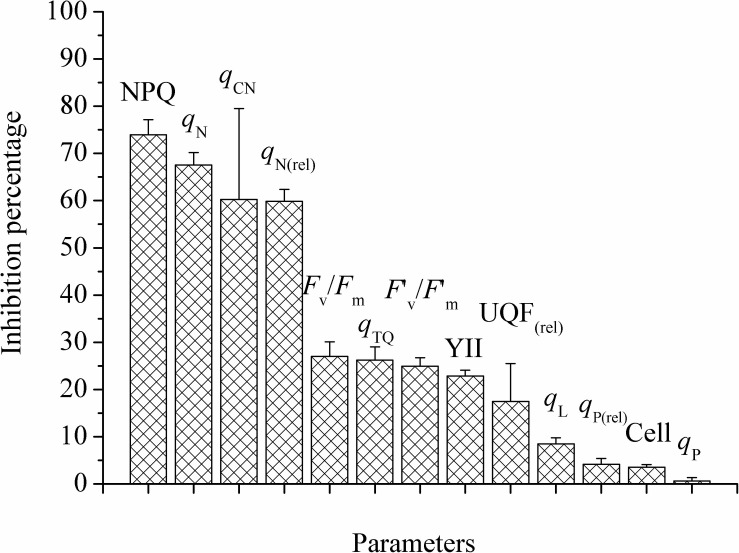
The inhibition percentages of different parameters with the mixture of PA and GA after 24 h for *M. aeruginosa*. The proportion of mixture was: PA: GA, 1:1. Values were means ± SD (*n* = 3). Cell means cell densities.

Figure 2 exhibited the inhibition percentage of PA-GA mixture on *M. aeruginosa* after 24 h. The inhibition percentages were more than 55% based on NPQ, *q*_N_, *q*_CN_ and *q*_N(rel)_, between 17.47 and 26.99% for *F*_v_/*F*_m_, *q*_TQ_, *F’*_v_*/F’*_m_, YII and UQF_(rel)_, and lower than 10% for *q*_L_, *q*_P(rel)_, cell densities and *q*_P_. It could be found that the sensitivity order of the parameters was non-photochemical quenching parameters > photochemical quenching parameters or others > cell densities. Based on all of the above results, NPQ and cell densities were selected for the following experiments.

### 72 h EC_50_ Values of the Different Polyphenols Mixtures Based on Parameters NPQ and Cell Densities

The 72 h EC_50_ values of the mixtures were listed in Tables 3, 4. Except for the mixtures of PA:CA/3:2 and PA:GA:CA/1:2:2, the 72 h EC_50_ values of *M. aeruginosa* based on NPQ were much lower than those of cell densities (*P* < 0.05). The EC_50 cells_ value was several times as lager as EC_50 NPQ_, and the highest times could reach 5.54 times in the mixture of PA:GA/4:1. Therefore, NPQ was more sensitive than cell densities. Except for PA:CA/3:2 and GA:CA/4:1, the EC_50 cells_ values increased with the increase of the low toxic compound (CA) ratio in the mixtures of PA-CA and GA-CA, but which was not found in the mixtures of PA-GA, and PA-GA-CA. The strongest inhibition was shown in the mixture of PA:GA/4:1 with EC_50 NPQ_ values of 0.46 ± 0.09 mg L^–1^, the mixture of GA:CA/1:4 presented the weakest inhibition with the EC_50 cells_ values of 9.84 ± 0.26 mg L^–1^. And they were nearly 21 times difference in EC_50_ values.

**TABLE 3:** The 72 h EC_50_ values (mg L^–1^) of the binary polyphenols mixtures on *M. aeruginosa* according to cell densities and NPQ at different proportions.

Mixtures	EC_50_ values				
PA:GA	1:4	2:3	1:1	3:2	4:1
Cell densities	3.17 ± 0.23^a^	4.05 ± 0.12^a^	3.47 ± 0.03^a^	4.13 ± 0.29^a^	2.55 ± 0.11^a^
NPQ	0.70 ± 0.18^b^	0.98 ± 0.18^b^	1.68 ± 0.35^b^	0.97 ± 0.25^b^	0.46 ± 0.09^b^
PA:CA	1:4	2:3	1:1	3:2	4:1
Cell densities	6.24 ± 0.18^a^	6.04 ± 0.37^a^	4.58 ± 0.19^a^	6.47 ± 0.48^a^	4.35 ± 0.34^a^
NPQ	1.56 ± 0.22^b^	1.40 ± 0.18^b^	2.68 ± 0.38^b^	5.81 ± 0.97^a^	2.09 ± 0.07^b^
GA:CA	1:4	2:3	1:1	3:2	4:1
Cell densities	9.84 ± 0.26^a^	5.74 ± 0.34^a^	5.15 ± 0.58^a^	4.24 ± 0.15^a^	6.10 ± 0.46^a^
NPQ	2.68 ± 0.39^b^	1.25 ± 0.26^b^	2.10 ± 0.40^b^	2.71 ± 0.53^b^	4.17 ± 0.35^b^

*Values were means ± standard deviation (n = 3). The significant differences were signed with different superscript letters (a,b) in the same column (P < 0.05).*

**TABLE 4:** The 72 h EC_50_ values (mg L^–1^) of the three polyphenols mixtures on *M. aeruginosa* according to cell densities and NPQ at different proportions.

Mixtures	EC_50_ values						
PA:GA:CA	1:1:3	1:2:2	1:3:1	1:1:1	2:1:2	2:2:1	3:1:1
Cell densities	5.66 ± 0.27^a^	7.70 ± 0.26^a^	6.35 ± 0.19^a^	5.70 ± 0.57^a^	7.63 ± 0.74^a^	5.48 ± 0.52^a^	6.62 ± 0.54^a^
NPQ	2.71 ± 0.49^b^	7.47 ± 0.95^a^	1.89 ± 0.04^b^	1.69 ± 0.30^b^	4.77 ± 0.80^b^	2.87 ± 0.57^b^	3.79 ± 0.43^b^

*Values were means ± standard deviation (n = 3). The significant differences were signed with different superscript letters (a,b) in the same column (P < 0.05).*

### Combined Effects Assessing by TI Mode

**FIGURE 3:**
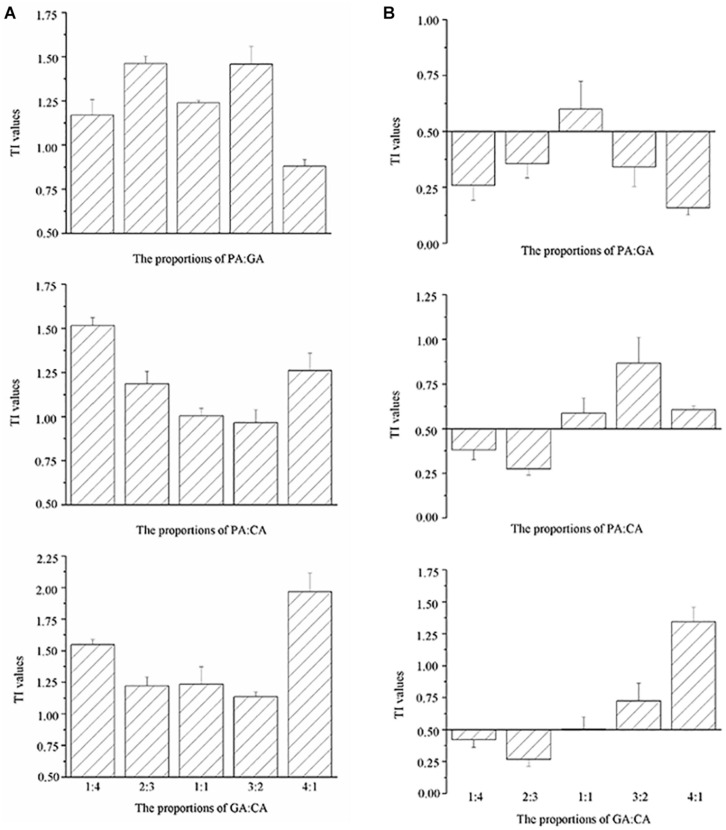
TI values calculated according to cell densities **(A)** and NPQ **(B)** of *M. aeruginosa* in the mixtures of binary polyphenols (means ± SD, *n* = 3).

**FIGURE 4:**
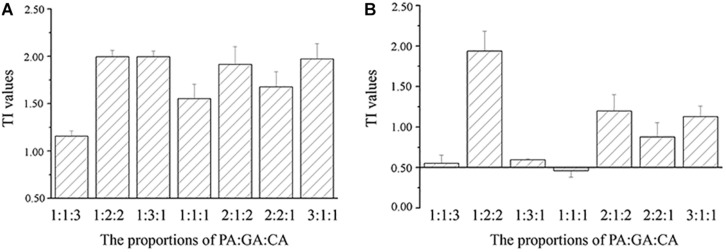
TI values calculated according to cell densities **(A)** and NPQ **(B)** of *M. aeruginosa* in the mixtures of three polyphenols (means ± SD, *n* = 3).

The TI values of the mixtures were reported on the histograms (Figures 3, 4). TI values based on the EC_50 cells_ values ranged from 0.88 to 1.99, which were included in the interval [0.5–2.0], revealing the additive effects of binary and three polyphenols on *M. aeruginosa* (Figures 3A, 4A). No correlation could be observed between TI values and the proportions of polyphenols in the mixtures. The TI values based on the EC_50 NPQ_ value varied from 0.16 to 1.94, covering the synergistic and cumulative effects (Figures 3B, 4B). Apart from the PA-GA/1:1, the mixture of PA-GA indicated a synergistic effect according to NPQ (Figure 3B). Other binary polyphenols presented synergistic effects at the ratio of 1:4 and 2:3 (Figure 3B). However, the three polyphenols only exhibited a synergistic effect at the ratio of 1:1:1 (Figure 4B). The synergistic effects of binary polyphenols were stronger than that of the three polyphenols by comparing their TI index (*p* < 0.05).

### Combined Effects Assessing by Isobolograms

**FIGURE 5:**
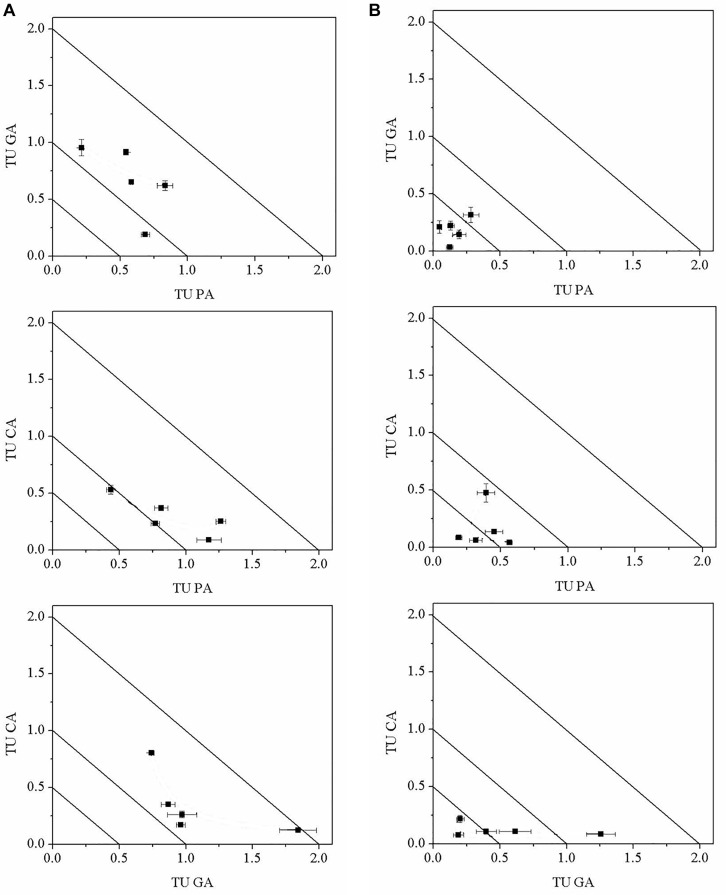
Isobologram representation according to cell densities **(A)** and NPQ **(B)** of *M. aeruginosa* in the mixtures of binary polyphenols (means ± SD, *n* = 3).

The TU values of the mixtures were shown on the isobologram (Figure 5). All the TU values based on the cell densities were between the straight lines X + Y = 2.0 and X + Y = 0.5, and the tested organisms were away from synergism and antagonism lines, revealing an additive effect of binary polyphenols on *M. aeruginosa* (Figure 5A). Figure 5B presented TU values of the binary polyphenols based on the EC_50 NPQ_ values of *M. aeruginosa*. The isobolographic plots revealed synergistic effects at the ratio of PA:CA/1:4, PA:CA/2:3, GA:CA/1:4, and GA:CA/2:3 on *M. aeruginosa*. For high toxic compounds PA and GA, except for the ratio of 1:1, isobolograms indicated synergistic effects for the most studied combination ratios. The results suggested binary polyphenols exhibited synergistic effects when the percentages of high toxic PA or GA lower than 40%. The results of isobolograms were in agreement with those of TI values.

## Discussion

Under environmental stress, the excess light energy may result in the formation of reactive oxygen species, which may irreversibly damage PSII and cause a continuous decline in photosynthetic efficiency (Weisz et al., 2017; Gao et al., 2018). The non-photochemical quenching parameters can be applied to estimate a share of energy dissipation within the antenna system, which is a self-protection mechanism of cells under excess light energy (Roach and Krieger-Liszkay, 2014; Xu et al., 2019). The excess light energy is eliminated by non-photochemical quenching to protect the normal progress of the photochemical reaction (Yong et al., 2018). Therefore, non-photochemical quenching parameters always increase to protect photosynthesis under mild stress or in the early stage of severe stress by quenching (e.g., pH-gradient build-up, LHC_2_ phosphorylation and zeaxanthin formation) and recovery (such as a stress-adapting D1 protein) mechanisms, but decrease with further damage (Cummings et al., 2018; Nowicka, 2019). Photochemical quenching parameters reflect the photochemical capacity of PSII in the light-adapted state and correspond to the steady-state fraction of oxidized PSII reaction centers (Zulfugarov et al., 2018). The decline of photochemical quenching parameters suggests the amount of energy used in photochemistry becomes small, namely, the actual photosynthetic efficiency is reduced (Campbell et al., 1998; Henriques, 2009). In this study, non-photochemical quenching parameters was the most sensitive parameters to PA and GA, followed by photochemical quenching parameters or others [*F*_v_/*F*_m_, *F’*_v_*/F’*_m_, *q*_TQ_, and UQF_(rel)_], and the least sensitive was cell densities. Under the stress of PA and GA, the PSII activity of *M. aeruginosa* was influenced at the beginning, but non-photochemical quenching could protect the photosynthesis (Sonani et al., 2018). The protective effects of non-photochemical quenching were gradually lost for further inhibition, and photochemical quenching parameters decreased followly, ultimatly, photosynthetic efficiency dropped significantly (Cummings et al., 2018). For instance, the expression of *psbA* gene encoding the stress-adapting D1 protein (the main components of PSII) was inhibited by PA in cyanobacteria *Cylindrospermopsis raciborskii* (Wu et al., 2013). The effect of environmental stress on photosynthesis was instant, but cell densities were a manifestation of cell growth and cell division, so cell densities lagged behind the response of chlorophyll fluorescence in time (Wang et al., 2016). However, for EA and CA, CFPs were not more sensitive than cell densities. This may be explained by the inhibitory site of PA and GA was PSII on *M. aeruginosa*, which was from the oxygen-evolving complex to the secondary PSII quinone electron acceptor (QB) and the entire electron transport chain; while EA and CA might show inhibitory effects on the photosynthesis by other mechanisms rather than photosynthetic system and the entire electron transport chain in toxicity bioassays, therefore, there is no obvious rule between the influence of CFPs and cell densities (Zhu et al., 2010; Wang et al., 2011). In return, the rule has been proved by PA-GA mixtures.

O_2_ and NAD(P)H were continuously produced through photosynthesis in *M. aeruginosa* cultured under light conditions (Hanelt, 2018). With the exposure of PA and GA, NAD(P)H was used as the electron donor to amplify the production of ROS by the pseudo-circular electron transport chain in photosynthesis and the ineffective redox cycle, which formed under inefficient electrons transfer within disrupted photosynthetic electron transport chain and might be greatly enhanced organisms toxicity, such as decrease of photosynthetic efficiency and cell growth rate, peroxidation of membrane lipid, destruction of cell structure and function, even cell death (Zhu et al., 2010; Wang et al., 2011; Gomes et al., 2017). However, the production of ROS was induced by the ineffective redox cycle for CA (Wang et al., 2011), and the EC_50_ value of CA was much higher than that of PA and GA as our research results show (2.97–25.80 times). So these might be the reasons for adding CA to the PA-GA mixtures did not change the sensitivity of NPQ. And similar results were also found in other researches, especially for monitoring photosynthesis inhibitors exposure in ecotoxicological test systems (Dewez et al., 2008; Macedo et al., 2008; Wang et al., 2016; Berg et al., 2017). CFPs were more sensitive than the growth during the effects of herbicides on macrophytes (Knauert et al., 2010). And CFPs served as the best markers for evaluating the quality of the edible blue-green algae *Nostoc flagelliforme* (Gao et al., 2014). Other CFPs such as YII, *q*_P_ and *q*_N_ could be used as early indicators of algae cells in response to heavy metals and fungicides (Lu et al., 2000; Dewez et al., 2005; Gan et al., 2019).

To study the action modes for highly effective inhibition of polyphenols with low concentrations on *M. aeruginosa*, two complementary methods, TI and TU models were used. The TI model is a quantitative evaluation of the combined effects between binary or multiple mixtures (Gao et al., 2015). In the combined effects of PA, GA and CA on *M. aeruginosa*, TI values showed different combined effects according to different parameter indicators. The combined effects of binary and multiple polyphenols based on cell densities indicated additive effects. However, the binary and three mixtures exhibited synergistic and additive effects on *M. aeruginosa* based on NPQ. Additionally, it was found that the synergistic effects of the two-dimensional combination of polyphenols were stronger than that of the three-dimensional combination by comparing their TI index. These may indicate that the combined effects of polyphenols were not only related to the species numbers of the mixtures, but also affected by other factors such as the property, proportion and concentrations of each substance in the mixtures (Zuo et al., 2016a,b). Of course, parameters chosen as evaluating indicators were important in the combined effects of polyphenols on *M. aeruginosa*, and suitable indicators might be helpful to reflect the mechanism of the combined effects (Wang et al., 2016). Compared with cell densities, CFPs, especially NPQ, were suitable parameters as evaluating indicators in the combined effects of polyphenols on *M. aeruginosa*. The TU model is a qualitative analysis of the combined effects of binary mixtures (Wilkinson et al., 2015). In this study, the results of the qualitative analysis were consistent with that of quantitative analysis.

Our research found that the combined effects of multiple polyphenol allelochemicals on *M. aeruginosa* were stronger than that of the single polyphenol treatment based on the sensitive response parameters NPQ. Although the inhibiting algae effects of the polyphenols with lower concentration could be explained partly by combined effects, allelopathy is complicated in natural habitats. The contents and types of polyphenols released by aquatic plants vary in different seasons and aquatic plants, which impacts the inhibitory activity of allelochemicals (Tazart et al., 2020). The environmental factors such as pH, light, and nutrition, also influence the effects of allelochemicals and sensitivity of target phytoplankton (Mohamed, 2017). The further research of these gaps is needed in the next step.

## Conclusion

(1) Chlorophyll fluorescence parameters (CFPs) presented more sensitive than cell densities. For PA and GA inhibiting photosystem II (PSII) activity of *M*. *aeruginosa* 905, the sensitivity order of parameters based on the EC_50_ values was: non-photochemical quenching parameters [NPQ, *q*_N_, *q*_N(rel)_ and *q*_CN_] > photochemical quenching parameters [YII, *q*_P_, *q*_P(rel)_ and *q*_L_] or others [*F*_v_/*F*_m_, *F’*_v_*/F’*_m_, *q*_TQ_ and UQF_(rel)_] > cell densities.
(2) The binary and three mixtures exhibited synergistic and additive effects on *M. aeruginos* based on NPQ, but additive effects based on the cell densities. According to NPQ, the synergistic effects were exhibited at binary polyphenols with a proportion of high toxic polyphenols (PA or GA) less than 40%, and three polyphenols only at the proportion of 1:1:1.
(3) The combined effects of polyphenols released by aquatic macrophytes with low concentration play an important role in inhibiting phytoplankton growth in natural aquatic ecosystems.

## Data Availability Statement

The raw data supporting the conclusions of this article will be made available by the authors, without undue reservation, to any qualified researcher.

## Author Contributions

SH and JZ conceived and designed the work, drafted the manuscript, and performed the experiments. BL revised the work critically for important intellectual content. LZ, XP, and XZ analyzed the results. FG and ZW revised the manuscript. All authors read and approved the final manuscript.

## Conflict of Interest

The authors declare that the research was conducted in the absence of any commercial or financial relationships that could be construed as a potential conflict of interest.

## Abbreviations

CFPs: chlorophyll fluorescence parameters
YII: the effective quantum yield of photochemical energy conversion in photosystem II (PSII)
*q*_P_: a parameter estimating the fraction of PSII centers in open states based on a puddle model for the photosynthetic unit
*q*_L_: a parameter estimating the fraction of PSII centers in open states based on a lake model for the photosynthetic unit
*q*_P(rel)_: relative photochemical quenching
NPQ: light induced photoprotection through thermal dissipation of energy in the antenna system
*q*_N_: light induced photoprotection through thermal dissipation of energy in thylakoid membrane
*q*_N(rel)_: relative non-photochemical quenching
*q*_CN_: complete non-photochemical quenching
*q*_TQ_: total quenching
*F*_v_/*F*_m_: the maximum quantum yield of PSII photochemistry
*F’*_v_*/F’*_m_: the effective quantum yield of PSII photochemistry
UQF_(rel)_: relative unquenched fluorescence
EC_50_: the higher 50% effective concentrations
TI: toxicity index
TU: toxic unit.
